# Mechanical and Thermal Properties of 3D-Printed Continuous Bamboo Fiber-Reinforced PE Composites

**DOI:** 10.3390/ma18030593

**Published:** 2025-01-28

**Authors:** Haiyu Qiao, Qian Li, Yani Chen, Yayun Liu, Ning Jiang, Chuanyang Wang

**Affiliations:** School of Mechanical and Electrical Engineering, Soochow University, Suzhou 215000, China; hyqiao@suda.edu.cn (H.Q.); qian0037@126.com (Q.L.); 20224029002@stu.suda.edu.cn (Y.C.); yyliu6688@suda.edu.cn (Y.L.)

**Keywords:** continuous natural fiber, bamboo fiber, 3D print, fiber–plastic interface, mechanical properties, thermal properties

## Abstract

Continuous fibers with outstanding mechanical performance due to the continuous enhancement effect, show wide application in aerospace, automobile, and construction. There has been great success in developing continuous synthetic fiber-reinforced composites, such as carbon fibers or glass fibers; however, most of which are nonrenewable, have a high processing cost, and energy consumption. Bio-sourced materials with high reinforced effects are attractive alternatives to achieve a low-carbon footprint. In this study, continuous bamboo fiber-reinforced polyethylene (CBF/PE) composites were prepared via a facile two-step method featuring alkali treatment followed by 3D printing. Alkali treatment as a key processing step increases surface area and surface wetting, which promote the formation of mechanical riveting among bamboo fibers and matrix. The obtained treated CBF (T-CBF) also shows improved mechanical properties, which enables a superior reinforcement effect. 3D printing, as a fast and local heating method, could melt the outer layer PE tube and impregnate molten plastics into fibers under pressure and heating. The resulting T-CBF/PE composite fibers can achieve a tensile strength of up to 15.6 MPa, while the matrix PE itself has a tensile strength of around 7.7 MPa. Additionally, the fracture morphology of printed bulks from composite fibers shows the alkali-treated fibers–PE interface is denser and could transfer more load. The printed bulks using T-CBF/PE shows increased tensile strength and Young’s modulus, with 77%- and 1.76-times improvement compared to pure PE. Finally, the effect of printing paraments on mechanical properties were analyzed. Therefore, this research presents a potential avenue for fabricating continuous natural fiber-reinforced composites.

## 1. Introduction

The outstanding mechanical performance of continuous fiber-reinforced composites, e.g., carbon fiber and glass fiber, shows wide application in aerospace, automobile, infrastructure, etc. [1,2,3], due to cost reduction and efficiency improvement. For example, a 10% reduction in vehicle weight by using composites can lead to a 6–8% improvement in fuel economy. Natural fibers, as one of the most abundant biomaterials on the Earth, possess low cost, high mechanical properties, and environmentally friendly properties [4,5], showing great potential in replacing carbon or glass fibers [6]. Taking bamboo fibers as an example, its tensile strength could reach 1.9 GPa [7], comparable to that of carbon fibers, while the cost is much lower than that of carbon fibers. Compared to the high temperature (>1000 K) and long process time required in fabricating carbon fibers [8] or glass fibers [9], the production process of the bamboo fibers features a low-carbon footprint, the processing cost and production energy of which is merely 8% and 3% of carbon fibers [10]. However, the practical application of continuous natural fiber-reinforced composites is restricted by the manufacture method. Therefore, developing a manufacturing method for fabricating continuous natural fiber-reinforced composites plays a critical role in expanding the application of natural fiber-reinforced composites.

The challenges lie in how to manufacture continuous natural fiber-reinforced composites without sacrificing their length. Traditionally, natural fibers, especially short fibers, are first mixed with thermoplastics using mixers and then pressed into desired shapes via injection or hot press machines [11]. Many natural fibers have been reported using the above-mentioned method, including bamboo fibers [12,13], jute fibers [14], flax fibers [15], and hemp fibers [16,17]. The strong shear force during the manufacturing steps contribute to the mixture of these natural fibers and thermoplastics, but it strongly shortens the length of the fibers [18]. For example, ramie fibers with the initial length of 5 mm decreased to 0.28 mm after melt compounding and injection molding, demonstrating that natural fibers are susceptible to breakage during the process [19]. Similar results have also been demonstrated in glass fibers [20] and carbon fibers [21]. In addition, the liquid molding process, known as resin transfer molding, has been reported for preparing natural fiber-reinforced composites. In the liquid molding process, the short bamboo fibers are obtained by chemical treatment and roller milling, then these fibers are compressed in the mold, and thermoset resins are impregnated into the empty space in the mold under pressure. Finally the composites are demolded after the resins cure [22,23]. Continuous bamboo fiber-reinforced epoxy composites have demonstrated outstanding mechanical properties with 2.8-times enhanced tensile strength and 5.24-times improved Young’s modulus compared to pure resin [24]. However, continuous bamboo fiber-reinforced thermoplastic composites is difficult to fabricate due to the higher viscosity, even in high temperature (nearly 3 orders of magnitude higher than that of resin [25,26]).

**Figure 1:**
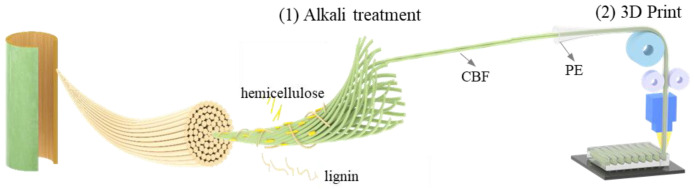
Schematic figure of CBF/PE composite using the proposed alkali treatment combined with 3D printing method.

This study proposes a facile two-step method to fabricate continuous natural fiber-reinforced composites (Figure 1). Bamboo fibers were chosen as the representative plant fibers considering the high grown rate and high mechanical performance. In this process, the natural bamboo fibers noted as untreated bamboo fibers (U-CBF) were first alkali-treated to partially remove lignin and hemicellulose content (the obtained treated bamboo fibers are abbreviated as T-CBF). Then, the T-CNF were fed into hollow PE tubes, serving as composite filaments. Finally, during the 3D printing process, the thermoplastic PE tube would melt and immerse into the center bamboo fibers under heating and pressure. The increased contact area and surface wetting after the alkali treatment step can promote the formation of mechanical riveting among bamboo fibers and PE tubes, leading to a dense and strong interface. This dense interface, along with long and strong treated bamboo fibers as reinforcements, contribute to substantially improved mechanical properties of composites. Note that proper process parameters are essential to avoid the nozzle clogging and guarantee the smooth extrusion during 3D printing.

## 2. Experimental Sections

### 2.1. Materials

Bamboo fibers were directly purchased from Mayu Town, Wenzhou (Zhejiang, China). The length and diameter of received fibers were 90 cm and 0.12–0.30 mm, respectively. It is worth noting that the length of the bamboo fiber is larger than that of traditional short and long fiber (around 0.2 mm and 2 mm, respectively). Here we defined them as continuous bamboo fibers. Sodium sulfite (Na_2_SO_3_) and sodium hydroxide were purchased from Sinopharm Chemical Reagent Co., Ltd. (Shanghai, China). PE tubes were purchased from Taizhou Baolilai plastic products factory (Taizhou, China). The outer and inner diameters of used PE tubes were 2 mm and 1 mm, respectively.

### 2.2. Preparation of Continuous Bamboo Fibers

The preparation process of bamboo fibers was as follows (Figure S1). First, the bamboo fibers were immersed in the mixed solution (2.5 M NaOH and 0.4 M Na_2_SO_3_) for 24 h at the temperature of 80 °C. The pH of the solution was 14.4 and the solution was mixed using magnetic stirring. Then, the bamboo fibers were rinsed with distilled water three times to remove the attachments. Finally, these bamboo fibers were dried in an oven at 75 °C for 12 h. These treated bamboo fibers were kept in the plastic vacuum dryer at room temperature.

### 2.3. Printing Composites Filaments and Bulks

The printing process was conducted on the fused deposition machine (T3040, Shenzhen Dakun 3D Technology Co., Ltd., Shenzhen, China). The parameters of the 3D printing settings are listed in Table 1.

### 2.4. Characterization

Tensile tests of samples were conducted on TY8000-A (Jiangsu tianyuan testing equipment Co., Ltd., Yangzhou, China) with a gauge length of 25 mm and a crossed rate of 2 mm/min. The test method used in this study references the preparation process of samples in previous reports [24]. Before tensile tests, the tested samples were attached on paper with epoxy adhesive and then dried at room temperature under pressure for at least 72 h to cure the adhere. During the tensile test, the two paper ends of samples were fixed on the stand. The tensile tests were repeated at least five times for each type of bamboo fiber, composite fiber, and printed bulks. Tensile stress, strain, Young’s modulus, and toughness were extracted from the tensile tests results. Tensile stress is determined by dividing the applied force by the original cross-sectional area of the sample when it was stretched until it breaks, and defined as:

*σ* = *F*/*A* (1)

where *F* is the applied force, and *A* represents the cross-sectional area.

Strain is calculated as the change in the length of the sample divided by its original length, and defined as:

(2) $$\varepsilon =\frac{l-l_{0}}{l_{0}}$$

where *l* represents the length upon fracture, and *l*_0_ is the original length.

Young’s modulus, which characterizes the material’s stiffness, is calculated by dividing the stress by the strain within the elastic range of the stress–strain curve, and defined as:

*E* = *σ*/*ε* (3)

Toughness is a measure of the material’s ability to absorb energy before fracture and is calculated by integrating the stress-strain curve from the origin to the breaking point, yielding the total area under the curve which represents the energy absorbed per unit volume of the material, and defined as:

(4) $$Toughness=\int_{0}^{\varepsilon_{f}}\sigma d\varepsilon$$

where *ε_f_* is the strain upon failure.

Scanning electron microscopy (SEM) was performed using the EVO 18 SEM (CARL ZEISS, Jena, Germany) operating at an accelerating voltage of 20 kV. X-ray diffraction analysis (XRD) analysis was conducted on D8 Discover PLUS, (Bruker, Dresden, Germany). The diffraction angle ranged from 5° to 35° at a scanning speed of 4°/min. Fourier transform infrared (FT-IR) analysis was conducted on a Nicolet iS20 (Thermo Fisher Scientific, Waltham, MA, USA). Thermogravimetric analysis (TGA) was conducted on a Q500 thermogravimetric analyzer (TA company, Boston, MA, USA) at a rate of 10 °C/min in nitrogen atmosphere from room temperature to 600 °C. Differential scanning calorimetry (DSC) analysis was conducted on Q2000 differential scanning calorimeter (TA company, Boston, MA, USA) at a rate of 10 °C/min in nitrogen atmosphere from room temperature to 350 °C. The software origin 2016 was used in this research to process the raw data and plot the figures.

## 3. Results and Discussion

### 3.1. Characterization and Performance Analysis of Alkali-Treated Bamboo Fibers

**Figure 2:**
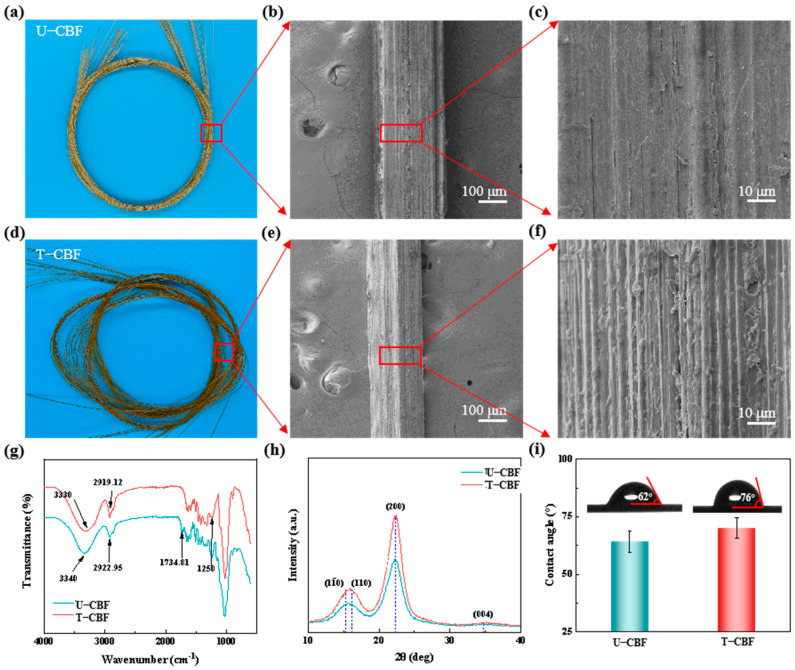
Characterization of bamboo fibers before and after the alkali treatment. The images, low and high magnification of SEM of (**a**–**c**) U-CBF and (**d**–**f**) T-CBF. (**g**) FT-IR spectra, (**h**) XRD, and (**i**) contact angle of U-CBF and T-CBF.

Figure 2 presents the morphology and microstructure of the bamboo fibers before and after alkali treatments. Figure 2a is the digital image of U-CBF, showing light yellow due to the existence of the lignin, hemicellulose, and other components. The SEM images in Figure 2b,c show the diameter is around 240 μm, and the surface is rough. Figure 2d presents the digital image of T-CBF, showing dark yellow due to the alkali treatment. The SEM image in Figure 2e reveals that the diameter of T-CBF decreased to 200 μm. Figure S2 presents the diameter changes of CBF with different diameters before and after the alkali treatment, revealing that the diameter reduction was approximately 18% compared to U-CBF. The enlarged SEM image in Figure 2f presents that the surface of the T-CBF is rougher, indicating more contact area with thermoplastics. Furthermore, Figure S3 presents the surface of bamboo fibers before and after the alkali treatment using the ultra-depth of field microscope. The quantitative results in Figure S4 show that the surface roughness of CBF increased from 6.98 μm to 11.01 μm after the alkali treatment. Both results demonstrate that the alkali treatment removed other components and exposed cellulose macrofibre bundles [27], which increased the roughness of the CBF, consistent with previous reports [22,28]. The exposed microfibers are aligned along the longitude direction, indicating a strong continuous reinforced effect. Fourier-transform infrared spectroscopy (FT-IR) was carried out to further confirm the compositional changes of the bamboo fibers after alkali treatment. As shown in Figure 2e, the peaks located at 1734 cm^−1^ are assigned to the functional groups of hemicelluloses (C=O stretching vibration), and the peaks locating at 1250 cm^−1^ are assigned to the functional groups of lignin (Guaiacyl-lignin and C=O stretching vibration). These results indicate significant removal of the two components after the alkali treatment, which is consistent with previous reports [5]. XRD results in Figure 2h reveal that the crystal peak position does not change, demonstrating that no phase transformation occurred during the alkali treatment. The crystal intensity increased and the width decreased, showing an improved cellulose crystallinity according to the Scherrer method [29]. This increased crystallinity indicates improved mechanical and thermal properties of T-CBF. Figure 2i shows the contact angles of U-CBF and T-CBF were 62° to 76°, separately. This could be attributed to the changes of the surface roughness and the polarity of the hydroxyl group after alkali treatments. The lignin would react with strong alkali due to the existing phenolic hydroxyl group and carboxyl and was removed after washing. The removed lignin reduces the polarity of hydroxyl groups of the whole fibers, resulting in the increase in the water contact angle. This increased contact angle indicates enhanced hydrophobic–hydrophobic interactions of bamboo fibers with plastics. The above characterizations demonstrate that bamboo fibers could serve as a strong reinforcement due to higher crystallinity and improved plastic–fiber interface.

**Figure 3:**
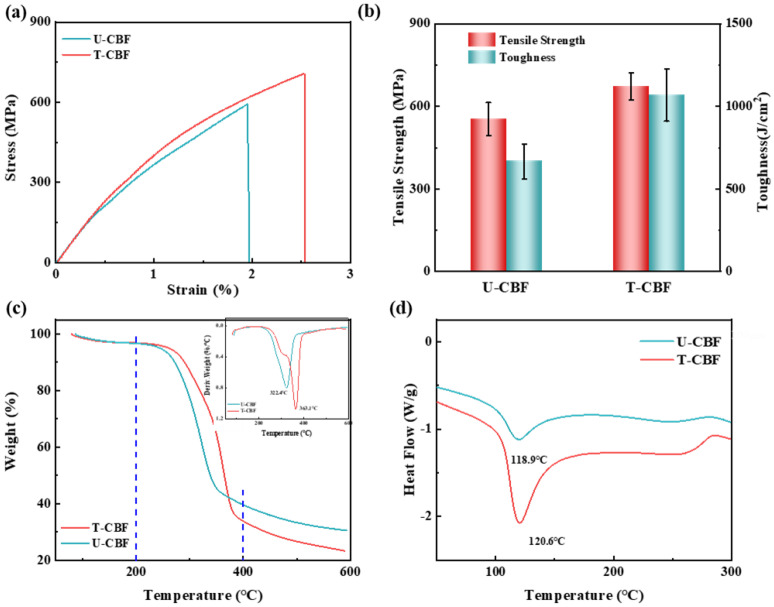
Mechanical and thermal analysis of the bamboo fibers. (**a**) tensile stress–strain curves and (**b**) tensile strength and toughness, (**c**) TG curves and (**d**) DSC curves of U-CBF and T-CBF. The inset figure in (**c**) are DTG curves.

Furthermore, the mechanical and thermal properties of bamboo fibers before and after alkali treatment were characterized. The representative stress–strain curves in Figure 3a reveals that both U-CBF and T-CBF share brittle fracture behaviors, while different from U-CBF, T-CBF shows improved mechanical properties evidenced by increased tensile strength, elongation, and Young’s modulus. The tensile strength of U-CBF and T-CBF were 553.7 ± 59.7 MPa and 672.3 ± 49.6 MPa, an increased of 21%, and the toughness was 666.3 J/m^3^ and 1067.2 J/m^3^, an increased of nearly 60% (Figure 3b). The Young’s modulus of T-CBF increased by 30% (Figure S5). The improved tensile strength could be attributed to the formation of a highly ordered continuous cellulose crystal structure due to the removal of impurities with lower mechanical performance, such as hemicellulose and lignin. As a result, the arrangement of the cellulose nanofibers in the crystal structure along the direction of tension could withstand more external load, presented as higher tensile strength. According to the rule of mixture, at the same content, the higher mechanical properties of the reinforcement, the stronger the mechanical performance of the composites. The increased toughness is related to the higher elongation and Young’s modulus. The higher elongation at break of T-CBF could be attributed to the produced nanovoids among cellulose nanofibers due to the removal of the hemicellulose and lignin after the alkali treatment. Those voids enable the relative movements of cellulose nanofibers under loading, increasing the plasticity of the CBF [30]. It should be noted that although the mechanical properties of the treated bamboo fiber are lower than the highest value reported in the studies [7], the value is still acceptable because the mechanical properties of plant fibers are highly relevant to the selected species, age, section, and tested size. The thermal properties were tested through thermogravimetric (TG) and DSC tests. As shown in Figure 3c, both TG curves of U-CBF and T-CBF show three stages, (1) losing the physically adsorbed water below 150 °C, (2) decomposing at the temperature range of 150–400 °C, and (c) a high temperature graphite zone above 400 °C. The corresponding derivative TG curves (DTG) is provided in the inset in Figure 3c, which reveals that the maximum weight loss temperature of T-CBF was higher than that of U-CBF by 40.7 °C. This is because the removed components including hemicellulose and lignin, are small molecules, which show a lower decomposition temperature compared to the remaining cellulose. The DSC curves in Figure 3d show that the melting temperatures of U-CBF and T-CBF were 118.9 °C and 120.6 °C, presenting a slight improvement in thermal stability.

### 3.2. Performance of 3D-Printed Continuous Bamboo Fiber-Reinforced PE Composite Filaments

**Figure 4:**
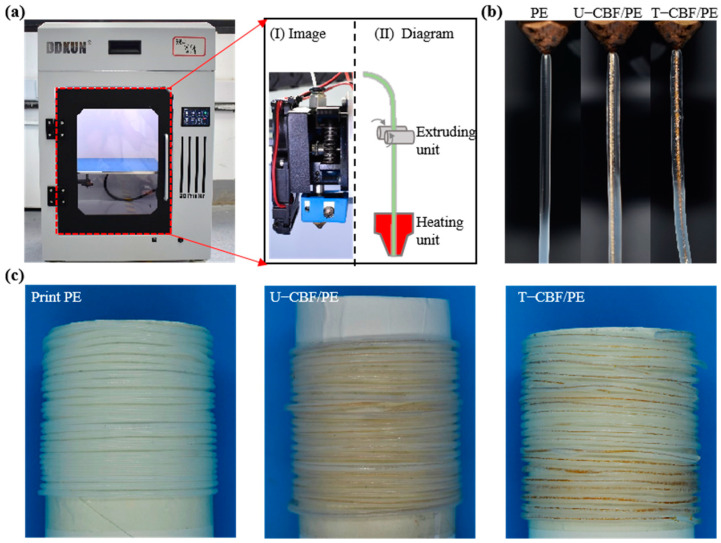
3D printing of continuous bamboo fiber-reinforced PE composite filaments. (**a**) Images of the 3D printer. The enlarged figures are images and schematic figures of the printing unit. (**b**) Images of printed composite fibers from the nozzle. (**c**) Images of printed filaments.

Figure 4a shows the digital image of the printing setup, where the outer plastics would first melt and then get impregnated into the fiber surface under the extruding unit and heating unit, as shown in the enlarged inset. To ensure the smooth extrusion of the material, a nozzle with a diameter of 0.8 mm and suitable printing parameters were selected. The heating temperature was 180 °C to form bamboo fiber-reinforced PE (noted as CBF/PE) composite material. Figure 4b exhibits the printed composite fibers through the printer, which shows that all the fibers, pure PE, U-CBF/PE, and T-CBF/PE, could print smoothly at suitable processing windows. Figure 4c shows the melting temperature of printed PE, U-CBF/PE, and T-CBF/PE composite fibers, demonstrating this method could fabricate the fibers in a large scale.

**Figure 5:**
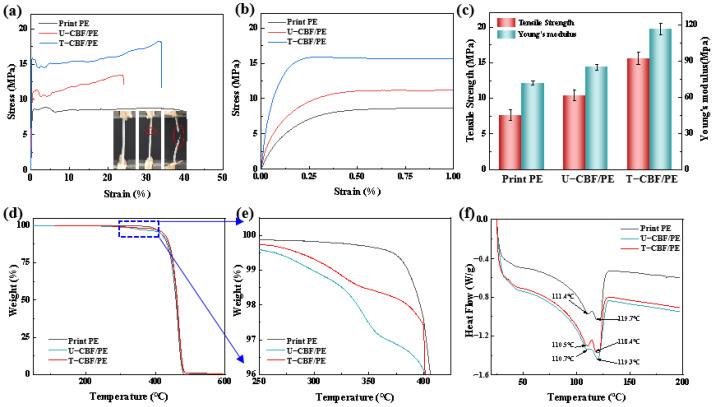
Performance of the composite fibers. (**a**) The stress–strain curves of fibers, inset: the images of the T-CBF/PE during tests. The red circles note the necking and fracture behaviour of composite fibers during testing; (**b**) the enlarged figure in (**a**); (**c**) the tensile strength, strain, and toughness of the composite fibers; (**d**) the TG curves of the composite fibers; (**e**) the enlarged figure in (**d**); (**f**) the DSC curves of the composite fibers.

The performance of composite filaments is fully characterized through tensile tests and thermal tests. The stress–strain curves in Figure 5a present that pure PE fibers and composite fibers all show three obvious zones, that is the linear elastic zone, yield behavior, and fracture. The inset figure shows T-CBF/PE shows an obvious necking before fracture. The elongation of T-CBF/PE composite fibers was 35%, and decreased by 15% compared to that of pure PE, and increased by 42% compared to UCBF/PE composite fibers. The impressive performance in elongation of T-CBF/PE could be attributed to the better interface between T-CBF and PE. The enlarged figure in Figure 5b shows that in the linear elastic zone, the bamboo fiber composites show a large slope, indicating a higher Young’s modulus. The quantitative comparison results in Figure 5c shows the treated composites present the following (1) improved tensile strength. The tensile strength value of pure PE, U-CBF/PE, and T-CBF/PE filaments were 8.6 MPa, 12.1 MPa, and 15.5 MPa. Table 2 shows statistical results of samples. Compared to pure PE, the tensile strength of CBF/PE and T-CBF/PE increased by 35% and 103%. (2) Increased Young’s modulus; compared to pure PE, the Young’s modulus of U-CBF/PE and T-CBF/PE increased by 19% and 62%.

TGA and DSC tests were performed to investigate the effect of alkali-treated fibers on the thermal properties of composite fibers. Figure 5d shows all fibers had three similar stages, verifying that adding fibers had a little effect on their thermal behavior. The enlarged figure in Figure 5e shows in the temperature range of 250–400 °C. Compared to U-CBF/PE, T-CBF/PE showed a slight increase due to the removal of the other components, indicating that alkali treatment does not affect the thermal stability of CBF/PE composite fibers. The DSC curves in Figure 5f show that the differences among these three fibers is less than 1 °C, indicating that adding fibers does not affect the melting temperature of the matrix. Note that the lower peak in the DSC curves of PE represents the plasticizer and additives to guarantee the smooth extrusion of PE materials.

**Figure 6:**
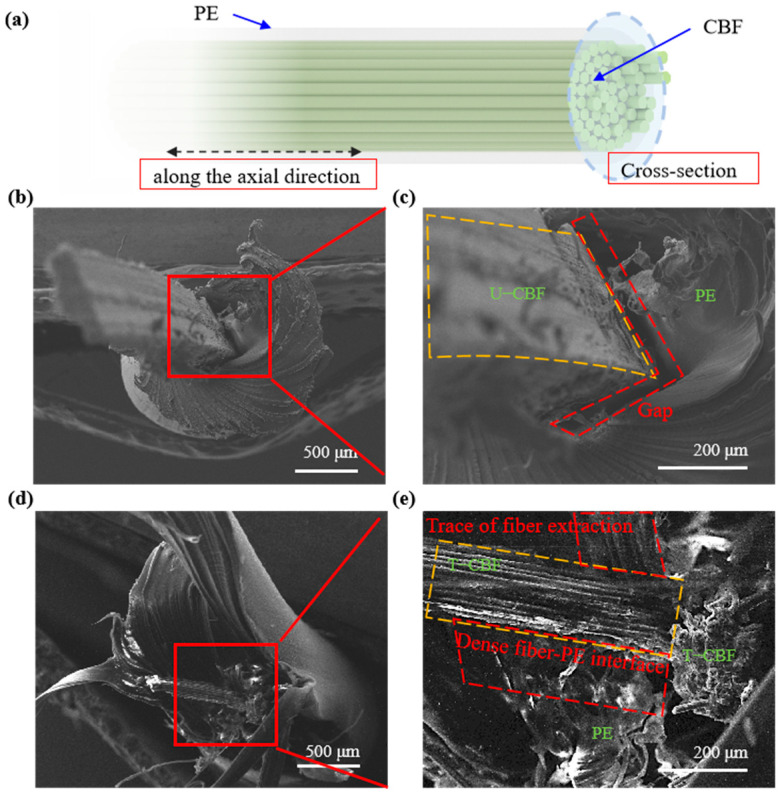
The SEM images of the cross-section of composite fibers after tensile tests. (**a**) The illustration of the observed direction. (**b**,**c**) U-CBF/PE composite fibers. (**d**,**e**) T-CBF/PE composite fibers.

Macroscopic images were obtained of the composite fibers after tensile tests were performed along and perpendicular to the direction of the fibers (Figure 6a shows the observation direction). The SEM images show that along the axial direction of fibers presented in Figure S6a,b the U-CBF/PE composite fibers present a clear peeling phenomenon. The enlarged SEM image in Figure S6c shows the peeled fiber is relatively smooth with a slight layer of adhesion plastics. The fracture morphology of the U-CBF/PE composite fibers in Figure S6d–f shows that the plastics were nearly fully impregnated into treated bamboo fibers, forming a tight contact interface. That is, the T-CBF/PE composite fibers show a tight coating effect of plastics on the treated bamboo fibers. Further observation on the cross-section of composite filaments is presented in Figure 6b–e. Note that the fiber would delaminate from the matrix after the tensile tests due to load transfer during the tests. Figure 6b shows that there is a large gap between U-CBF and PE melt in the composite filaments after tensile fracture, and only a very small amount of PE melt bonded to U-CBF in the enlarged image (Figure 6c). Both showed poor bonding performance between U-CBF and PE, indicating the interface is easily separated during the tensile testing process. Figure 6d,e show that the gap at the pulled end face is small, and peel off traces (traces of fiber extraction are highlighted in the figure) could be obviously observed at the PE area. The irregular fracture of the fibers shows the PE immersed into fibers at micro-level, which indicates a better bonding performance. This strong interface could serve as a bridge to efficiently transfer load from the matrix to fibers during tests, exhibited as improved mechanical properties. The interfacial forces among the matrix–plant fiber plays an important role in the properties of the composites, so, surface chemical treatment of plant fibers will further enhance their adhesion to the matrix [31], thereby improving the mechanical properties of composite materials.

### 3.3. Performance and Morphology Analysis of the Printed Composite Bulks

**Figure 7:**
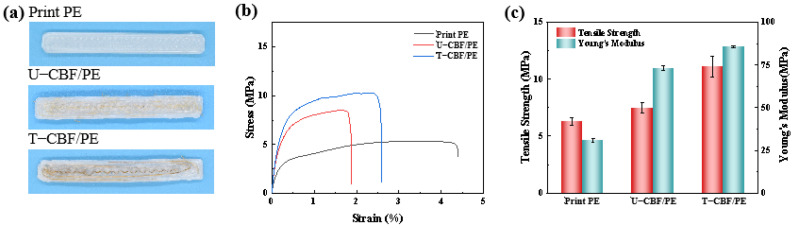
The mechanical performance of the bulks printed using different composite fibers. (**a**) The digital images of the printed bulks. (**b**) The stress–strain curves, and (**c**) the tensile strength and Young’s modulus of the printed bulks.

Figure 7 shows the images and the tensile results of the printed bulks from pure PE, U-CBF/PE, and T-CBF/PE. The printing speed was 5 mm/s, the printing layer thickness was set as 0.3 mm, and the hot plate temperature was 60 °C. Figure 7a presents the images of the printed bulks. Compared to pure PE bulks, the bamboo fibers were distributed within bulks with fibers. Note that some untreated bamboo fibers fractured and stretched outside while the treated bamboo fibers were distributed in the bulks without fracture according to the preset printing route. This could be attributed to the relative brittleness of U-CBF, as demonstrated in Figure 3b, which is prone to curling or breaking under high extrusion temperature and high pulling when the fibers extrude from the nozzle and deposit into the platform. Figure 7b,c show the tensile properties of the obtained composite bulks. Compared with the printed bulks of pure PE, the tensile strength of the bulks with fibers increased but the strain decreased. Furthermore, bulks strengthened by T-CBF showed increased tensile strength and strain compared to bulks of U-CBF. The obvious improvement in tensile performance of bulks of the treated fibers might result from the continuous strengthening effect from CBF. The details of the tensile properties of the bulks are shown in Table 3 and plotted in Figure 7c. The tensile strength of PE, U-CBF/PE, and T-CBF/PE bulks were 6.3 ± 0.3 MPa to 7.5 ± 0.5 MPa and 11.1 ± 0.9 MPa. That is, compared to pure PE bulks, the tensile strength of U-CBF/PE and T-CBF/PE bulks increased by 19% and 77%. The Young’s modulus of the bulks shared a similar increased pattern. The Young’s modulus of PE, U-CBF/PE, and T-CBF/PE bulks was 31.0 ± 1.0 MPa, 73.2 ± 1.3 MPa, and 85.8 ± 0.6 MPa. Compared to pure PE bulks, the Young’s modulus of U-CBF/PE and T-CBF/PE bulks increased by 1.36-times and 1.76-times.

**Figure 8:**
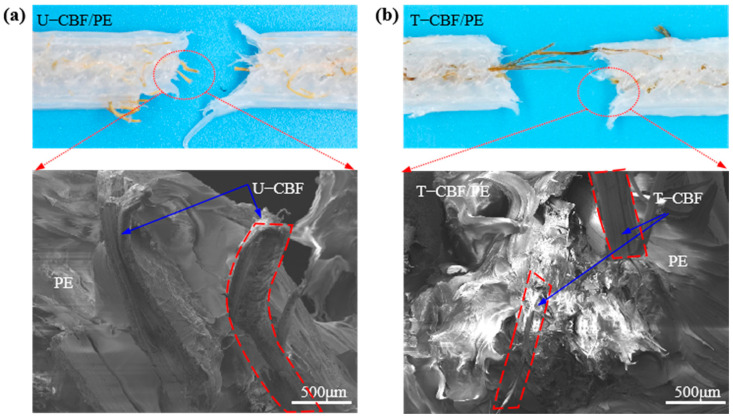
The digital and SEM images of (**a**) U-CBF/PE and (**b**) T-CBF/PE.

Furthermore, the morphology of the fracture section is presented in Figure 8. Figure 8a shows fewer fibers occur at the cross-section when the composite bulks were prepared by U-CBF/PE fracture according to the digital image. The corresponding SEM image shows (1) U-CBF are pulled out in the whole with little residual fibers; (2) the interface between U-CBF and PE is smooth. Both indicate the load is less transferred to the fibers. Figure 8b shows that more stretched fibers occur at the cross-section for the composite bulks prepared by T-CBF/PE from digital images. The corresponding SEM image shows that the interface between T-CBF and PE is vague, and a small portion of the expenditure fibers noted by the arrows are pulled out or buried in PE, which means the fiber–matrix interface could better transfer load. The above analysis demonstrates this strong adhesion is conducive to the continuous reinforcement effect on the PE material.

### 3.4. Effect of Printing Parameters on Tensile Performance of Bulks

**Figure 9:**
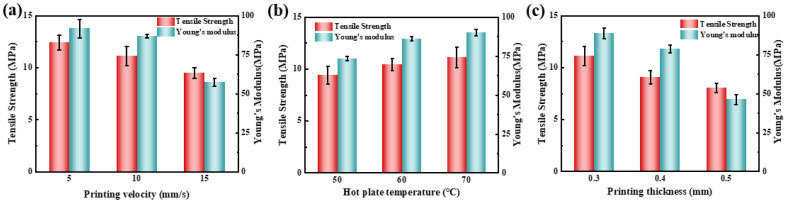
The mechanical performance of the bulks printed using T-CBF/PE composite fibers under different printing parameters. (**a**) Velocity, (**b**) temperature, and (**c**) thickness.

Compared with traditional composite materials made by hot pressing, the problem of being unable to “compact” in this layer-by-layer stacking method of 3D printing could result in low bonding performance and high porosity between layers. Therefore, the influence of various printing parameters on the mechanical properties of composite materials during the printing process was studied. The main printing parameters included printing velocity, hot plate temperature, and layer thickness. It should be noted that fiber content also plays an important role in the performance of the composites, but adding more fibers into tubes would result in nozzle clogging during printing, so the effect of fiber contents was not taken into account in this study. Figure 9 shows the mechanical properties of bulks by T-CBF/PE composite fibers under different printing parameters, and the details are listed in Table 4. Figure 9a reveals that with the increase in the printing velocity, the tensile strength of T-CBF/PE composite bulks decreases. This is because when the printing velocity increases, the filament and the molten plastics are both stretched, resulting in decreased contact area. Excessive printing velocity may even cause the breakage of bamboo fibers, thereby affecting the forming quality of printed splines. In addition, an increase in printing velocity shortens the immersion time of molten PE into T-CBF. This would induce a negative impact on the interface bonding between T-CBF and PE, resulting in ineffective load transfer from PE material to T-CBF during stretching of the sample and a decrease in tensile strength. Figure 9b shows that with the increase in hot plate temperature, the tensile strength of T-CBF/PE bulks slightly increases. Increasing the hot plate temperature reduces the temperature gradient between the deposited layer and the extruded filament, which is conducive to the bonding between these layers. It is worth noting that because the temperature of the hot plate has a greater impact on the bonding performance between the bottom layer and the neighbor layer and a smaller impact on the bonding performance of the upper layer, the improvement of the hot plate temperature on the tensile strength of the composite bulk is limited. Figure 9c shows that as the layer thickness increases, the tensile strength of the composite bulks obviously decreases. As the layer thickness increases, the heat affected area between the layers decreases [32]. That is, increasing the layer thickness causes an increase in the height and a decrease in the width of the deposited filaments [33], resulting in poor bonding quality due to the less contact area. Other defects like pores may also likely appear between adjacent filaments. The larger deposition pressure from the smaller layer thickness during heating would strengthen the immersion effect of the T-CBF into PE, achieving better adhesion by avoiding the defects at the interface and increasing density. A smaller layer thickness also corresponds to less extruded PE melt, increasing the content of T-CBF and improving the uniformity of fiber distribution in the matrix, thereby reducing the occurrence of defects, and ultimately improving the tensile strength of printed splines.

## 4. Conclusions

In this study, we have demonstrated that a simple two-step method, alkali treatment combined with 3D printing, could fabricate continuous bamboo fiber-reinforced PE composites. The alkali treatment removes lignin and hemicellulose, thereby increasing the surface area, contact angle, mechanical, and thermal properties, making T-CBF a better reinforcement. The 3D printing step induces a rapid impregnation of matrix into fibers under heat and pressure. As a result, we achieved a dense interface between T-CBF and PE within composite filaments, revealed by SEM images. Such a dense interface contributes to an improved tensile strength, and Young’s modulus, which are 103%, and 62% higher than that of pure PE. Moreover, the printed T-CBF/PE bulks show increased tensile strength and Young’s modulus, with 77% and 1.76-times improvement compared to those of pure PE. The printing parameters analysis demonstrated that the high-performance mechanical properties of the printed bulks could be achieved by lower printing velocity, high bed temperature, and smaller layer thickness. We anticipate that the proposed alkali treatment and 3D printing method, not only improve the mechanical and thermal properties of the bio-composites, but also provide a technique to develop various continuous plant fiber composites, thus allowing the broader community in the field of transportation and infrastructure to explore and benefit from high-performance bio-composites. Improvements including upgrading experimental setups to add more fibers, and introducing additives will be conducted to further optimize the performance of the composites.

## Figures and Tables

**Table 1:** The parameters of 3D printing settings.

Parameter	Unit	Value
Printing speed	mm/s	5, 10, 15
The diameter of the nozzle	mm	0.8
Temperature of the nozzle	°C	190
Temperature of the hot plate	°C	50, 60, 70

**Table 2:** The mechanical properties of printed PE, U-CBF/PE, and T-CBF/PE filaments.

	Tensile Strength/ MPa	Young’s Modulus/MPa
Print PE	7.7 ± 0.7	71.8 ± 1.8
U-CBF/PE	10.4 ± 0.8	85.1 ± 2.5
T-CBF/PE	15.6 ± 0.8	116.5 ± 4.9

**Table 3:** The mechanical properties of printed PE, U-CBF/PE, and T-CBF/PE bulks.

	Tensile Strength/MPa	Young’s Modulus/MPa
Print PE	6.3 ± 0.3	31.0 ± 1.0
U-CBF/PE	7.5 ± 0.5	73.2 ± 1.3
T-CBF/PE	11.1 ± 0.9	85.8 ± 0.6

**Table 4:** The mechanical properties of T-CBF/PE bulks at different printing parameters.

Parameters	Value	Tensile Strength/MPa	Young’s Modulus/MPa
Velocity/mm/s	5	12.4 ± 0.7	91.8 ± 6.0
	10	11.1 ± 0.9	86.8 ± 1.1
	15	9.5 ± 0.5	57.4 ± 2.6
Temperature/°C	50	9.4 ± 0.8	73.4 ± 1.3
	60	10.5 ± 0.6	86.2 ± 1.5
	70	11.1 ± 0.9	90.2 ± 2.1
Thickness/mm	0.3	11.1 ± 0.9	88.6 ± 3.4
	0.4	9.1 ± 0.6	78.8 ± 2.5
	0.5	8.1 ± 0.5	46.3 ± 3.2

## Data Availability

The original contributions presented in this study are included in the article/supplementary material. Further inquiries can be directed to the corresponding authors.

## Supplementary Materials

The following supporting information can be downloaded at: https://www.mdpi.com/article/10.3390/ma18030593/s1, Figure S1: The fabrication process of the T-CBF; Figure S2: The diameter changes of the T-CBF after the alkali treatment; Figure S3: The surface of the U−CBF (a,c) and T-CBF (b,d) observed using ultra-depth of field microscope; Figure S4: The surface roughness of the U-CBF and T-CBF; Figure S5: The Young’s modulus of the U-CBF and T-CBF; Figure S6: The SEM images of the axial direction of composite fibers after tensile tests. (a–c) U-CBF/PE composite fibers. (d–f) U-CBF/PE composite fibers.
