# Supplementary material for: Mechanical and Thermal Properties of 3D-Printed Continuous Bamboo Fiber-Reinforced PE Composites
Source: Materials (Basel). 2025 Jan 28;18(3):593. doi: 10.3390/ma18030593 (PMC11818552; doi:10.3390/ma18030593)
Supplement: Supplementary file 1 [file materials-18-00593-s001.zip › materials-3422901-supplementary.pdf]

Supporting information

Article

Mechanical and Thermal Properties of 3D-Printed Continuous Bamboo Fiber-Reinforced PE Composites

Haiyu Qiao, Qian Li, Yani Chen, Yayun Liu, Ning Jiang * and Chuanyang Wang *

School of Mechanical and Electrical Engineering, Soochow University, Suzhou 215000, China; hyqiao@suda.edu.cn (H.Q.); qian0037@126.com (Q.L.); 20224029002@stu.suda.edu.cn (Y.C.); yyliu6688@suda.edu.cn (Y.L.)

* Correspondence: jiangning@suda.edu.cn (N.J.); cywang@suda.edu.cn (C.W.)

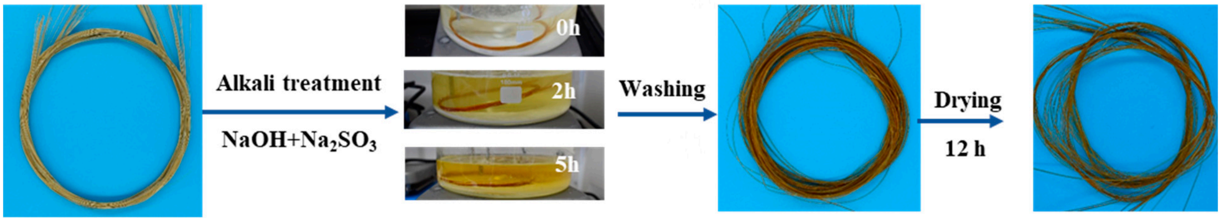

Figure S1. The fabrication process of the U-CBF

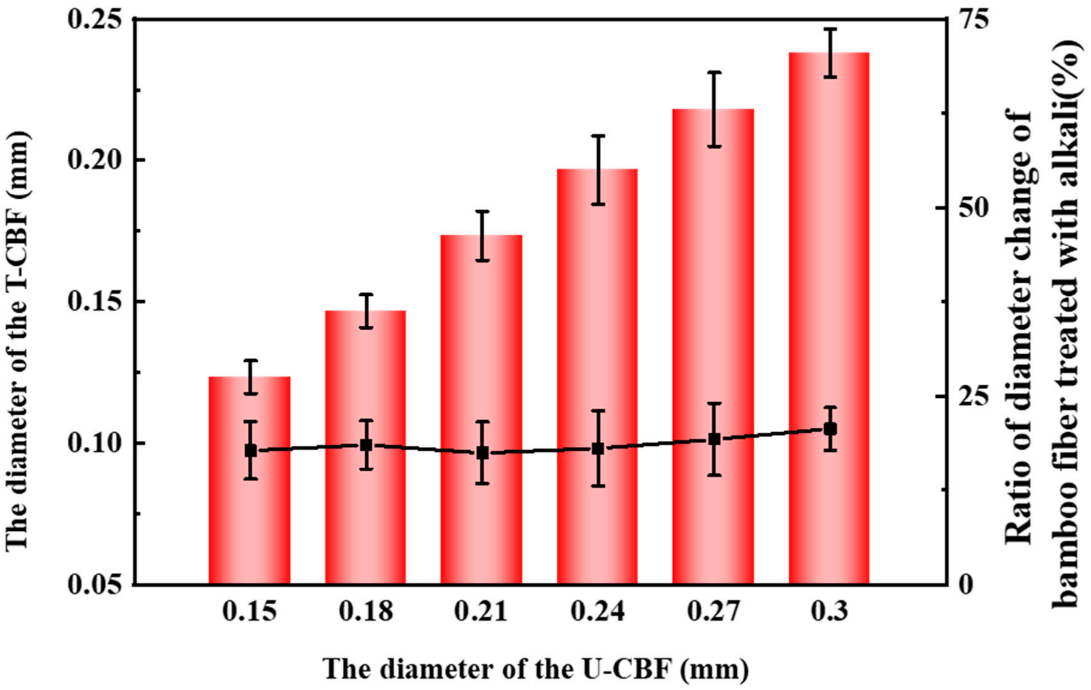

Figure S2. The diameter changes of the T-CBF after the alkali treatment

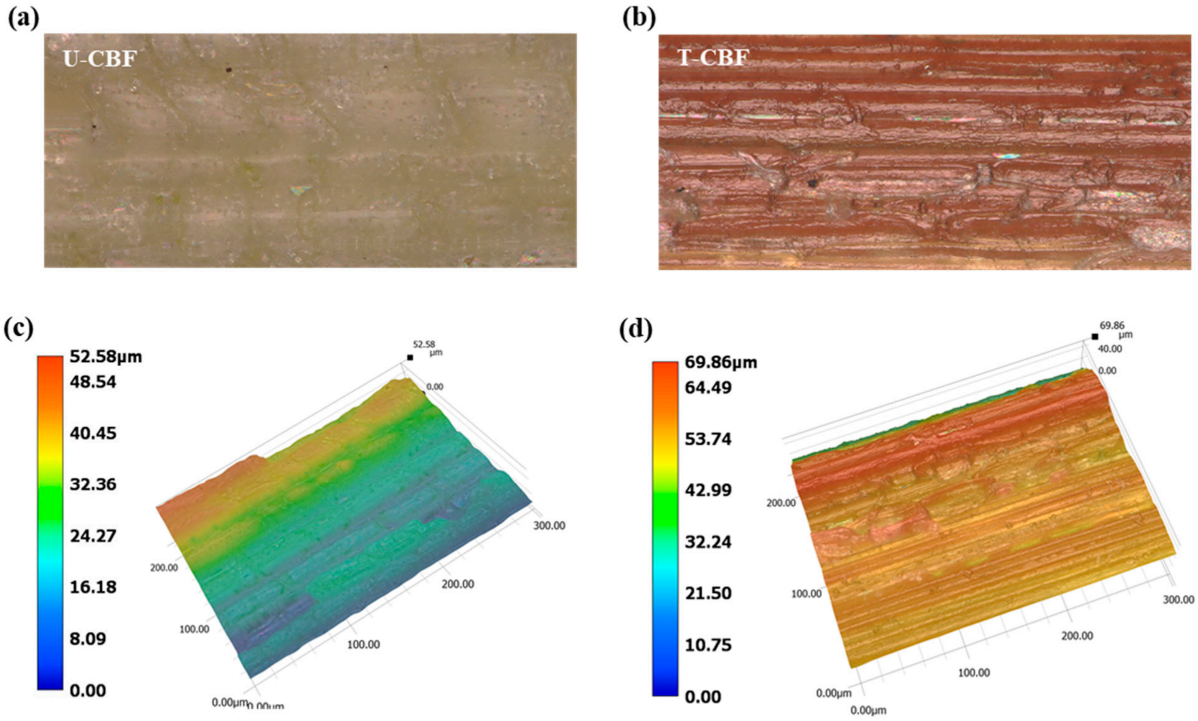

Figure S3. The surface of the UCBF (a,c) and T-CBF (b,d) observed using ultra-depth of field microscope.

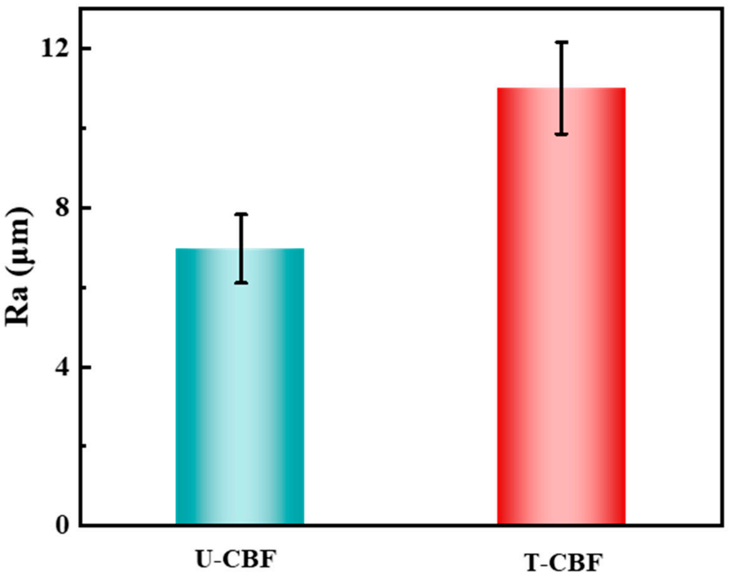

Figure S4. The surface roughness of the UCBF and T-CBF

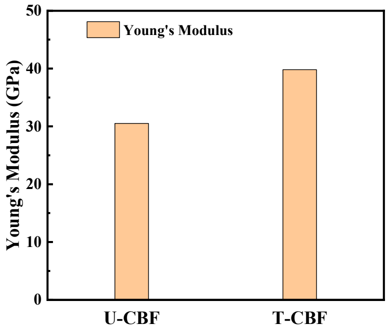

Figure S5. The Young's modulus of the UCBF and T-CBF

Figure S6 presents the macroscopic image of the composite fibers after tensile tests. Figure S6a observed in the direction of along the fiber shows the U-CBF/PE composite fibers, presents a clear peeling phenomenon. The enlarged SEM image in Figure S6b-c show the peeled fiber is relative smooth with a slight layer of adhesion plastics. Figure S6d shows the fracture morphology of the U-CBF/PE composite fibers. Figure f shows that the plastics is nearly fully impregnated into treated bamboo fibers, forming a tight contact interface. That is, the T-CBF/PE composite fibers shows a tightly coating effect of plastics on treated bamboo fibers.

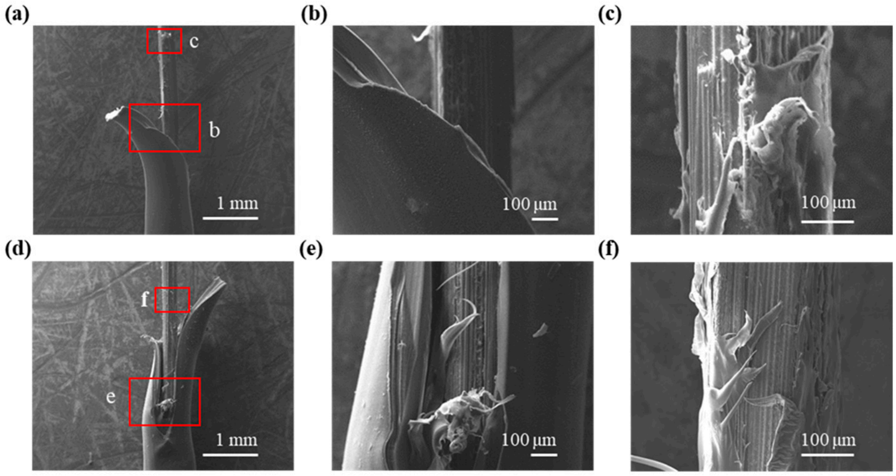

Figure S6. The SEM images of the axial direction of composite fibers after tensile tests. (a-c) U-CBF/PE composite fibers. (d-f) U-CBF/PE composite fibers.
